# Concurrent Associations between Expressive Language Ability and Independence in Adolescents and Adults with Fragile X Syndrome

**DOI:** 10.3390/brainsci11091179

**Published:** 2021-09-08

**Authors:** Leonard Abbeduto, Jessica Klusek, Julie Lounds Taylor, Nadia Abdelnur, Nicole Sparapani, Angela John Thurman

**Affiliations:** 1 MIND Institute & Department of Psychiatry and Behavioral Sciences, University of California, Davis, CA 95817, USA; nabdelnur@ucdavis.edu (N.A.); ajthurman@ucdavis.edu (A.J.T.); 2 Department of Communication Sciences and Disorders, Arnold School of Public Health, University of South Carolina, Columbia, SC 29208, USA; klusek@sc.edu; 3 Department of Pediatrics, Vanderbilt University Medical Center, Nashville, TN 37232, USA; julie.L.taylor@vumc.org; 4 MIND Institute & School of Education, University of California, Davis, CA 95616, USA; njsparapani@ucdavis.edu

**Keywords:** expressive language, fragile X syndrome, independent functioning, daily living skills

## Abstract

Background. Few individuals with fragile X syndrome (FXS) successfully meet adult normative expectations in education, employment, peer relations, and habitation, although there is within-syndrome variability in this regard. The primary goal of this study was to determine whether expressive language skills contribute to the capacity for independent functioning in adulthood even after controlling for nonverbal cognitive ability. Methods. Participants were 18- to 23-year-olds with FXS. Expressive language was assessed using the psychometrically validated Expressive Language Sampling (ELS) conversation and narration procedures. The language produced was transcribed and analyzed to yield measures of expressive vocabulary, syntax, and intelligibility. Parents concurrently completed questionnaires on the independent functioning of the participants with FXS. Results. All three ELS measures were significantly corelated with multiple measures of independence. The magnitudes of the correlations were reduced when nonverbal IQ was controlled through partial correlation. Nonetheless, many of the partial correlations were medium to large and several were statistically significant. Conclusions. Expressive language skills appear to contribute uniquely to the capacity for independence, although longitudinal data are needed to evaluate the possibility of a bidirectional relationship between these domains. Thus, language intervention may be a prerequisite for preparing youth with FXS for an independent adult life.

## 1. Introduction

### 1.1. Fragile X Syndrome

Fragile X syndrome (FXS) is the leading inherited cause of intellectual disability (ID), with a prevalence of 1 in 3600 males and 1 in 4000 to 6000 females [1,2,3]. The syndrome is caused by a mutation in the *FMR1* gene on the X chromosome and involves an expansion of a repetitive sequence of trinucleotides (CGG; [4]). This expansion leads to methylation of the gene and a consequent absence or reduction in the fragile X mental retardation protein (FMRP)—a protein that is critical for experience-dependent neural development [5].

In addition to ID, the phenotype associated with FXS is defined by especially serious challenges in multiple domains of functioning. In the cognitive domain, the greatest challenges are observed in attention [6], executive function [7], memory [8], and language [9,10]. The FXS phenotype is also characterized by co-occurring mental health concerns, including elevated rates (relative to the general population) of anxiety [11,12], hyperarousal [13,14], and hyperactivity [15,16]. There is also a high cooccurrence of autism spectrum disorder (ASD), particularly in males, who as a group have a prevalence of ASD of 50% or greater [17,18,19].

Although there is a “typical” profile of relative challenges and strengths associated with FXS, there is considerable within-syndrome heterogeneity. Males with the full mutation are more severely affected than females, for example, because the latter have a second unaffected X chromosome and, thus, higher levels of FMRP [20]. More than 90% of males meet criteria for ID, whereas only about one-third of females meet criteria, although females often have more circumscribed cognitive challenges, such as executive function problems [21,22,23]. Phenotypic variation, however, is related not only to sex but also to (within-sex) FMRP levels, additional background genes, and environmental influences [24].

### 1.2. Independent Functioning and Expressive Language in Adulthood

The array of challenges defining FXS is lifelong, and individuals with FXS are dependent on varying degrees of support even as they transition into adulthood. Indeed, only a minority of young adults successfully meet normative expectations in education, employment, peer relations, and habitation [25,26]. At the same time, however, there is evidence of within-syndrome variability in the capacity for functioning independently. Hartley et al. [26] examined independence relative to normative expectations for adults across a range of contexts, including vocational and leisure activities, using a survey of parents and caregivers. Among males with FXS, 90% displayed a very low to moderate level of independence, whereas about 1% received the highest rating for full independence. Among females with FXS, 56% were characterized as being in the very low to moderate range of independence, whereas 20% received the highest rating for independence. Variation related to age has also been observed in some studies, with improvements in independent functioning seen across adolescence and at least into the early adult years [26,27] (but see Dykens et al. [28,29] for exceptions). More severe symptoms of ASD appear to be associated with a less well-developed capacity for independent functioning [30]. Unfortunately, with the exception of ASD symptom severity, the factors promoting and inhibiting the capacity to function independently have not been adequately explored for FXS.

Similar to FXS, many individuals with nonsyndromic ASD have been found to experience especially severe challenges in daily living skills and other adaptive behaviors that limit independence in adolescence and adulthood [30,31,32]. In contrast to FXS, there have been several longitudinal studies that have examined predictors of outcomes in the adolescent and adult years for individuals with ASD. In these studies, several significant predictors have been identified, including IQ [33] and early motor skills [34]. Arguably, however, the most consistent predictor of independence in the adult years is the display of spoken, or expressive, language early in development, particularly evidence of “meaningful” speech (i.e., spontaneous, nonimitative use of a reasonably sized vocabulary) before the age of 5 years [35,36,37]. This relationship reflects, in part, the fact that individuals with stronger language skills are more apt to participate in the types of learning and social activities over the course of development that will better prepare them for adulthood [38,39]. It is also likely, however, that early language skills are predictive of language skills later in adulthood, and stronger language skills in adulthood will support greater concurrently measured independence for adults with ASD. Such concurrent relationships, however, have seldom been examined [40].

In light of the symptom overlap between FXS and nonsyndromic ASD [41,42,43,44], it is reasonable to hypothesize that expressive language ability will be positively correlated with the concurrently measured level of independent functioning in youth with FXS as they transition into adulthood as well; however, empirical data regarding this relationship are limited. At the same time, however, it is important to recognize that language is not a unitary ability; instead, language consists of several conceptually distinct but related components, or dimensions (e.g., syntax and vocabulary), which are likely to be differentially challenging for individuals with FXS and perhaps differentially related to an individual’s level of independent functioning. Examining the relationships between independent functioning and multiple dimensions of language is thus critical for identifying the language skills to be targeted in educational and transition programs designed to support greater independence among youth with FXS as they move into adulthood. The aim of the present study was to examine these concurrent relationships for older adolescents and young adults with FXS. In doing so, we assessed dimensions of spoken language likely to shape the capacity for independent functioning and did so using Expressive Language Sampling (ELS), which has recently been psychometrically validated for use in FXS [45,46,47] and other ID conditions [48,49].

### 1.3. Present Study

The research questions and hypotheses follow:

1. Are expressive language skills related to concurrently measured levels of independence for adolescents and young adults with FXS? It was hypothesized that stronger expressive language skills as assessed by ELS procedures would be associated with greater levels of independent functioning.
2. Do expressive language skills make a unique contribution to independent functioning over and above the contributions of development in other domains (e.g., nonverbal cognition) in adolescents and young adults? Although language development is itself shaped in important ways by achievements in cognition and other domains, it was hypothesized that expressive language skills would make a unique contribution to concurrently measured independent functioning.

## 2. Materials and Methods

### 2.1. Participants

Participants for this project were drawn from a larger longitudinal study investigating language development in transition-age adolescent and young adults with FXS (R01HD024356). Participants were recruited nationally through electronic advertisements distributed by the National Fragile X Foundation, the participant registries of the Intellectual and Developmental Disabilities Research Centers of the UC Davis MIND Institute (P50HD103526) and the Carolina Center for Developmental Disabilities (P50HD103573), and through cohorts previously developed by the PIs and their colleagues at each site. Participants for the present study were enrolled and assessed in person at either the UC Davis MIND Institute or the University of South Carolina. The parent who identified as the primary caregiver of the individual with FXS also participated and completed all the caregiver report measures included in the present study; in the present study, the participating parent was always the mother of the individual with FXS.

Participants met the following inclusion/exclusion criteria at enrollment. (1) The participant with FXS had been previously diagnosed with the *FMR1* full mutation (with or without mosaicism). (2) The participants with FXS were in their final year of high school according to parent report, which could be grade 12 or the final year of participation in a school-based transition program for youth with ID. Note that the “final year” was operationalized as extending for the summer before to up to six months after anticipated program exit, thereby including the summer months. (3) The parent indicated that the individual with FXS used speech as the primary means of communication, used some spontaneous three-word or longer phrases, and had no serious (uncorrected) sensory/physical impairments. (4) At the first annual assessment (Time 1), the individual with FXS lived at home with the participating parent. (5) The participant with FXS and the participating parent were fluent English speakers, with English as the primary language of the home, which was necessary because of the lack of non-English versions of most of the measures used in the study. Institutional Review Board approval was obtained at the participating university sites. Written consent was obtained from the parents and oral assent from the participants.

We included in the present study only those participants from the larger project who completed both the conversation and narration procedures and were assessed before the COVID-19 pandemic. Thus, all participants were tested in person at one of the university sites without the use of masks or other personal protective equipment. These criteria led to a sample of 24 adolescents and young adults with FXS (19 males). The characteristics of the sample are presented in Table 1. The sample was predominantly male, white, and non-Hispanic/Latinx. All but two participants (1 male, 1 female), had IQ scores that were consistent with a diagnosis of ID (i.e., 75 or below, to account for the margin of error). The range of family incomes was quite broad but was skewed toward those with incomes well above the median income for the U.S., which is USD 78,500 [50]. The participants with FXS ranged in age from 18 to 23.

### 2.2. Measures

The measures on which we report here are a subset of a larger battery of direct assessments, questionnaires, and interviews from the longitudinal project. The full battery is administered over the course of one or two days depending on the availability and stamina of the individual with FXS. The measures for this study were the ELS procedures, a standardized measure of cognitive ability, and several measures designed to collectively capture multiple dimensions of independent functioning through parent report.

#### 2.2.1. Expressive Language Sampling

Expressive language samples (ELS) were collected in two contexts—conversation and narration—from each participant. Manuals describing ELS administration, training, and the assessment of fidelity are available at https://ctscassist.ucdmc.ucdavis.edu/ctscassist/surveys/?s=W9W99JLMNX (accessed 7 September 2021).

The conversation task [45] consists of a 12-min interview-style interaction with the examiner. Examiners rely primarily on open-ended prompts to topics (e.g., “Tell me everything you did at school yesterday), use broad follow-up questions and prompts (e.g., “What do you like about school?”), and try to minimize their own talk. In addition, the examiner introduces a predetermined set of topics in a scripted order. The goal is to introduce at least three topics in addition to an initial idiosyncratic topic. Additionally, a minimum of one or two follow-up prompts are attempted for each topic before moving on to the next topic. If the list of topics is exhausted prior to reaching 12 min, the examiner can introduce up to two additional idiosyncratic topics of interest to the participant as reported by the parent. Two alternate versions of the conversation task were administered, with different topics in each. Half of the participants received version A and half version B.

The narration task [45] consists of the participant telling the story depicted in a wordless picture book. The examiner introduces the activity and asks the participant to look at each page spread of the book for 10 s without talking so as to gain a sense of the story. The examiner controls the page turning. The participant then tells the story page by page, with the examiner waiting 5 to 7 s until the participant has finished talking before turning the page. The examiner’s prompts and responses are standardized and limited largely to the first page of the book. There is no set time limit for the narration task. Two books, each including 16 pages of story content, from the Mercer Mayer’s “Frog” series were used: *Frog Goes to Dinner* (Version A) and *Frog on His Own* (Version B). Half of the participants received version A and half version B.

Conversation was always administered before narration, and each participant completed other measures between the two ELS procedures. All ELS sessions were digitally audiorecorded and the first 10 min transcribed and analyzed using SALT: Systematic Analysis of Language Transcripts [51]. All transcripts were prepared by a primary transcriber and reviewed by a secondary transcriber before being finalized. Transcribers were blind to individual participant results for other measures. Inter-transcriber agreement was randomly assessed for four transcripts (two conversation and two narration) from 4 different participants and found to have an average agreement of 92% across relevant dimensions of the transcription process.

Talk was segmented into Communication-units (C-units); the upper bound of which is an independent clause and any modifiers. We focused on the three ELS outcome measures shown to have the strongest psychometric properties across multiple studies and samples of individuals with FXS [45,46], computing each separately for conversation and narration and then averaging across the two tasks. The measures were:

Lexical Diversity. This measure indexes the size of the participant’s expressive vocabulary and is operationalized as the number of different word roots in 50 complete and fully intelligible C-units (or the full sample of complete and fully intelligible C-units if the participant produces fewer than 50 C-units). Higher scores indicate more advanced expressive vocabulary.

Syntax. This measure provides a gross measure of expressive syntactic competence and is computed as the mean length of C-unit measured in morphemes (MLU) for complete and fully intelligible C-units. Higher scores indicate more advanced expressive syntax.

Unintelligibility. This measure provides an index of speech articulation problems and is computed as the proportion of the total C-units that are either partly or fully unintelligible in the transcript. Higher scores indicate more problems with articulation.

#### 2.2.2. Cognitive Ability

The Stanford–Binet Intelligence Scales, Fifth Edition [52] Abbreviated Battery (SB-5/AB) was administered to the participants with FXS. This battery consists of the Vocabulary and Object Series/Matrices subtests, with the former indexing Verbal Knowledge (VK) and the latter indexing Nonverbal Fluid Reasoning (NFR). The SB-5/AB yields an abbreviated IQ with a mean of 100 in the norming sample and a standard deviation of 15. As floor effects are common for the SB-5 when used with individuals with ID, we used the deviation IQ scores developed by Sansone et al. [53], which are based on a transformation of the *z*-scores for the general population norms. The SB-5/AB deviation IQ data are presented in Table 1. We used the *z*-scores for NFR subtest in the analyses designed to determine whether expressive language skills predict concurrent independent functioning after controlling for the contribution of cognitive ability.

#### 2.2.3. Independent Functioning

The *Waisman Activity of Daily Living Skills* (W-ADL; [54]) is a caregiver report measure. The W-ADL was chosen because it was specifically designed to assess the daily living skills of adolescents and adults with ID [54], has been used successfully to characterize daily life skills in adolescents and adults with ASD [31,32,55], and provides a different indicator of this domain relative to previous research focused on validating the ELS measures on individuals with FXS [47]. The W-ADL indexes skills via 17 items distributed across the following domains: caring for self/personal daily living skills (6 items); caring for home/domestic daily living skills (9 items); and living in the community/community daily living skills (2 items). Parents rated the individual’s status for each skill as “independent,” “does with help,” or “does not do at all.” These descriptive ratings are assigned a score of 2, 1, and 0, respectively. We used the total score in all analyses, with a higher score reflective of greater independence and the maximum being 34. Note that the W-ADL was shown to have strong psychometrics for a large sample of adolescents and adults with various developmental disabilities, including those with FXS. In particular, Cronbach’s alpha was near 0.90 for a single factor solution; weighted kappas for reliability over time exceeded 0.90; correlations with other measures of daily living skills, such as the VABS-2 screener, were significant and near 0.80; and scores were significantly associated with several indicators of degree of impairment [54,56].

The *Vineland Adaptive Behavior Scales-3* (VABS-3; [57]) is a caregiver report measure of three broad domains of adaptive behavior—Communication, Daily Living Skills, and Socialization—with each domain comprised of three subdomains. The Comprehensive Interview version of the VABS-3 was used. The measure was normed on individuals ages from birth to 90 years, including individuals with ID. Due to the significant heterogeneity observed in the cognitive abilities of males and females with FXS, participants started at the mental age scored derived from the SB-5. In the present study, we used growth scores for the three Socialization subdomains: Interpersonal Relationships, Play and Leisure, and Coping Skills. Growth scores are on an interval scale and are designed to reflect an individual’s absolute level of ability. Growth scores range from 10 to near 130 depending on the subdomain, with higher scores reflecting greater ability. We focused on the three subdomains rather than the superordinate Socialization domain because, unexpectedly, Shaffer et al. [47] did not find any significant correlations of that domain with the ELS measures for their full sample of participants. We were interested in evaluating the possibility that there are differential patterns of associations with the ELS measures that were obscured by Shaffer et al.’s use of the Socialization domain score. The VABS-3 has strong psychometrics, including significant correlations with earlier versions of the VABS and the ABAS-3 and test–retest reliabilities between 0.55 and 0.72 for the three subdomains of interest for adolescents and adults.

The *Social Participation Index* (SPI) is a caregiver report measure about the adolescent or adult’s frequency of participation in social, recreational, and leisure activities (e.g., spends time with relatives he/she does not live with). Each of the 14 questions is rated from 0 (less than yearly or never) to 4 (several times a week). We used the total score, with a higher score indicating more frequent participation in the queried activities and the maximum score being 56. The SPI is based on the National Survey of Families and Households as adapted by Orsmond et al. [58]. In terms of psychometrics, there is evidence that the SPI score correlates significantly with a measure of friendship for adults on the autism spectrum (*r* = 0.395) and is predicted by severity of social impairments (*B* = −219), services received (*B* = 0.162), and degree of inclusion in school (*B* = 0.162), thereby providing evidence of construct validity [58].

The *AIR Self-Determination Scale* (AIR-SDS) is a caregiver report measure [59]. The scale is designed to profile the youth’s level of self-determination and to identify strengths and areas needing improvement. The scale was field-tested with students between the ages of 6 and 25 years. There are two components to the scale: (1) capacity, defined by the individual’s knowledge, abilities and perceptions and (2) opportunity, defined by the individual’s chances to use their capacity. The scale indexes skills via 18 items across the following categories: “things my child does” (6 items), “what happens at home” (6 items) and “what happens at school” (6 items). Each item is labeled by the parent for a five-point scale ranging from 1 (never) to 5 (always). Lastly, the scale seeks open-ended responses to three questions: (1) an example of a goal the child is currently working on, (2) what the child is doing to reach that goal, and (3) how the child is doing in reaching the goal. We used the total score, with a higher score indicating greater levels of self-determination. There is evidence of strong internal consistency (i.e., split-half reliability of 0.95) and test–retest reliability (i.e., 0.74 over a three-month interval) when completed by educators [59], although there do not appear to be comparable data from parents. Note that this measure was added to the protocol of the larger longitudinal study partway through the first wave of data collection; thus, AIR-SDS scores were available only for 12 of the participants in the present sample.

#### 2.2.4. Statistical Analysis Plan

We computed descriptive statistics for the ELS and independent functioning measures. We also examined the variables and their residuals for all correlations for the assumptions of normality required in the parametric tests used, applying appropriate transformations when assumptions were violated. To address the first research question, we computed Pearson correlations between each ELS measure (derived from averaging the scores for conversation and narration) and each measure of independent functioning. To address the second research question, we computed partial correlations between the ELS variables and measures of independent functioning controlling for chronological age and the SB-5/AB NFR deviation score. In each set of analyses, one-tailed inferential tests were used in all cases because we had clear hypotheses about the directionality of the relationships of interest. Familywise alpha levels for the correlations and for the partial correlations were maintained at *p* ≤ 0.050 levels through application of Benjamini and Hochberg’s false discovery rate [60] procedures.

## 3. Results

### 3.1. Descriptive Statistics

Means and standard deviations are presented for the ELS measures and the measures of independent functioning in Table 2 and Table 3, respectively. The ELS unintelligibility measure and the VABS-3 Coping Subscale growth score each deviated substantially from a normal distribution and, thus, a square root and logarithmic transformation, respectively, were applied to achieve normality. The untransformed scores are presented in the tables, however, to facilitate interpretation.

### 3.2. Primary Analyses

As seen in Table 4, the ELS measures were significantly correlated with the functional measures of independence, with better performance on the ELS measures associated with greater levels of independent functioning. Indeed, the only nonsignificant correlation was between the ELS unintelligibility measure and the VABS-3 Play subdomain score. The significant correlations were medium to large in magnitude according to the conventions established by Cohen [61]. These correlations remained significant even after controlling for multiple comparisons with the FDR procedure.

Partial correlations between the ELS and independent functioning measures controlling for chronological age and nonverbal cognitive ability (i.e., SB-5/AB NFR deviation IQ) are presented in Table 5. Far fewer of the partial correlations reached statistical significance relative to the simple zero-order bivariate correlations in Table 4; however, all three ELS measures correlated significantly with at least two measures of independence at an individual alpha level of 0.050 or better. After application of the FDR procedure, however, only three partial correlations remained significant, two involving syntax and one involving unintelligibility (see Table 5). At the same time, however, the magnitude of two nonsignificant correlations in Table 5 exceed +/−0.50, which Cohen [61] suggested was “large” in terms of strength of association. Thus, a total of 5 of the 18 partial correlations are large according to the Cohen guidelines, and these correlations include all three ELS measures.

In computing the partial correlations, we found that the correlations of age with the ELS and independent functioning measures were consistent with our recruitment strategy. In particular, older age was associated with less skill as individuals graduating high school at the end of Grade 12 were, on average, less impaired than those remaining in school to participate in a transition program. In contrast, and as would be expected, higher SB-5/AB NFR deviation IQs were positively correlated with more skill on the ELS and independent functioning measures.

## 4. Discussion

The goal of this study was to begin addressing the lack of data available on the role of expressive language skills in shaping the ability of individuals with FXS to function independently as they leave high school and school-based transition programs to face the normative tasks of young adult life. We found that all but one of the 18 bivariate correlations between the ELS measures and the parent-reported indices of independent functioning were significant. This pattern of findings is consistent with the hypothesis that more advanced language skills allow increased independence, as reflected in mastery of daily living skills, meeting social expectations, coping with novel situations, participating in the community, and being able to make one’s own important life decisions. It is also likely, however, that there are bidirectional relationships among the constructs of interest, such that opportunities for independence create opportunities for practicing and acquiring new expressive language skills. Although the concurrent nature of the present data does not allow us to address these bidirectional possibilities, the data clearly establish the important link between expressive language and the capacity for independence and suggest a need for further research including longitudinal data.

We also found that controlling for the contributions of age and nonverbal cognitive ability substantially reduced the magnitude of the correlations between the ELS measures and the measures of independent functioning. Nevertheless, 7 of the 18 partial correlations between the ELS measures and measures of independent functioning were statistically significant at an individual alpha level of 0.05 and five of these were large in terms of the effect size represented. Lastly, three partial correlations involving the ELS syntax and unintelligibility measures remained significant after correcting for multiple comparisons. Thus, our findings indicate that problems in intelligibility and limited expressive syntax are associated with a less well-developed capacity for independence in adolescents and young adults with FXS, and that these dimensions of expressive language make a contribution over and above their levels of skill and maturity in other domains. Expressive vocabulary, however, may not make a unique contribution to independent functioning.

In interpreting the partial correlations, however, it is also important to acknowledge the very strong correlations we observed between nonverbal IQ and the ELS measures, which makes it difficult to assess their separate contributions to independent functioning. Indeed, given the strong relationship between language and cognition, our partial correlations likely provide a conservative estimate of the contributions of expressive language ability to independent functioning. The relative contributions of language and cognitive abilities to the capacity for independence, therefore, should be explored in future research with an expanded range of measures of language, cognition, and independent functioning, which would also necessitate a larger sample of participants. Such research is needed given the highly verbal nature of the normative tasks of adulthood, such as securing and maintaining employment, making friends and establishing romantic relationships, navigating in the geography of the community, learning to manage finances, and gathering information to make important life choices such as where to live. Future research should also explore longitudinal as well as concurrent relationships among language, cognition, and independent functioning to identify potential routes for intervention to improve quality of life as youth with FXS transition to adult life. It would also be important to examine receptive language contributions to independent functioning.

Although we focused exclusively on individuals with FXS, the present findings suggest a need to better understand the factors that contribute to the capacity for independence in other conditions associated with intellectual and developmental disabilities. The symptom overlap between FXS and nonsyndromic ASD in particular suggests a need to build on previous research examining associations between early language and later functional outcomes in ASD. There is a need to also understand the ways in which language skills during and after the transition from school are related to the capacity for independence in individuals with ASD. Moreover, both short-term longitudinal and concurrent associations should be examined during the adolescent and young adult years.

The present study is characterized by several limitations that should be noted. First, the sample size is relatively small, especially for analyses involving the AIR Self-Determination Scale. Larger samples would provide more statistical power for detecting the strengths of associations among constructs as well as increased confidence in the generalizability of the findings. It will be important in future research to also include more objective measures, such as indicators of employment and residential circumstances, rather than relying solely on parent reporting. Finally, as noted previously, we relied on concurrently administered measures, which make it impossible to unambiguously determine the direction of causality in the relationships observed. Establishing a potential causal link between language and independence will require simultaneously examination of both concurrent and longitudinal associations.

## 5. Conclusions

The present study makes an important contribution to the literature on the late adolescent and young adult period of development in individuals with FXS, the leading inherited cause of intellectual disability. In particular, the present findings linking expressive language and independent functioning raise the possibility that interventions that lead to improvements in expressive language, especially in speech intelligibility and expressive syntax, may lead to, or at least provide a foundation for, a more independent adult life for those with FXS. Unfortunately, there are few such evidence-based language interventions for this population and those that do exist seem to be most beneficial for improving expressive vocabulary rather than intelligibility and syntax [62,63,64,65]. There is thus a pressing need to develop interventions that target these other dimensions of expressive language.

This study also addresses the call for psychometrically sound measures for evaluating treatment efficacy in studies of individuals with ID [66,67,68]. In particular, we have shown previously that ELS-derived measures are feasible, are subject to only minimal practice effects, have strong test–retest reliability, and have construct validity for individuals with FXS and other forms of ID [45,49]. The data from the present study show that differences on ELS-derived measures are associated with real-world functional competence in late adolescence and early adulthood, which is an association valued by treatment-regulating bodies, such as the U.S. Food and Drug Administration, when deciding on the utility of an outcome measure for establishing treatment efficacy. It is hoped that future treatment studies will begin to incorporate ELS measures and avoid selecting measures simply for ease of administration, such as clinician or parent ratings of perceived improvement, or measures that, while objective and reproducible, such as eyetracking, may have weak or unknown links with meaningful functional outcomes for individuals with ID.

## Figures and Tables

**Table 1:** Characteristics of Participating Youth with FXS.

Measure	M	SD	Range
Chronological Age (years)	20.48	1.50	18–23
SB-5/AB Deviation IQ	42.91	24.26	6.42–93.06
Distribution of Participants			
Race White—21 Asian—1 Multi-racial—1 Unknown—1	Ethnicity Hispanic/Latinx—2 Non-Hisp/Latinx—21 Unknown—1	Sex Female—5 Male—19	Family Income ^a^ USD 35,000–60,000—3 USD 60,001–150,000—6 USD 150,001–250,000—4 USD 250,001–300,000 or >6

*n* = 24 unless indicated otherwise. ^a^ *n* = 19.

**Table 2:** Means and Standard Deviations for the ELS Measures.

Measure	Conversation		Narration		Combined ^a^	
	**M**	**SD**	**M**	**SD**	**M**	**SD**
Syntax	4.64	2.36	6.22	3.20	5.43	2.65
Lexical Diversity	99.71	43.55	86.38	46.57	93.04	43.00
Unintelligibility ^b^	0.11	0.12	0.12	0.19	0.11	0.14

*n* = 24. ^a^ Average of conversation plus narration. ^b^ Untransformed scores presented, but transformed scores used in analyses.

**Table 3:** Means and Standard Deviation for Measures of Independent Functioning.

Measure	M	SD
W-ADL ^a^	22.68	5.58
VABS-3 Intrprsnl Play Coping	111.54 110.54 77.33	14.59 29.63 20.47
AIR-SDS ^a^	58.85	13.18
SPI ^b^	23.91	10.69

*n* = 24 unless noted otherwise. ^a^ *n* = 22, ^b^ *n* =12.

**Table 4:** Pearson Correlations Between ELS Measures and Independent Functioning Measures.

Measures	W-ADL ^a^	VABS-3 Intrprsnl	VABS-3 Play	VABS-3 Coping	SPI ^a^	AIR-SDS ^b^
Lexical Diversity	0.73 *****	0.46 *	0.59 ****	0.50 **	0.42 *	0.70 **
Syntax	0.70 *****	0.60 ****	0.49 **	0.55 ***	0.43 *	0.78 ***
Unintelligibility	−0.70 *****	−0.60 ****	−0.34	−0.52 ***	−0.49 **	−0.67 **

*n* = 24 unless otherwise noted. Shaded cells are significant at *p* ≤ 0.050 after FDR correction. * *p* ≤ 0.050, ** *p* ≤ 0.010, *** *p* ≤ 0.005, **** *p* ≤ 0.001, ***** *p* ≤ 0.0005. All tests one-tailed. ^a^ *n* = 22; ^b^ *n* = 12.

**Table 5:** Partial Correlations Between ELS Measures and Independent Functioning Measures Controlling for Chronological Age and SB-5/AB Nonverbal Knowledge Deviation IQ.

Measures	W-ADL ^a^	VABS-3 Intrprsnl	VABS-3 Play	VABS-3 Coping	SPI ^a^	AIR-SDS ^b^
Lexical Diversity	0.33	0.41 *	0.20	0.02	0.32	0.59 *
Syntax	0.36	0.60 ***	0.11	0.23	0.34	0.74 **
Unintelligibility	−0.40 *	−0.60 ****	0.15	−0.16	−0.39 *	−0.51

*n* = 24 unless otherwise noted. Shaded cells are significant at *p* ≤ 0.050 after FDR correction. * *p* ≤ 0.050, ** *p* ≤ 0.010, *** *p* ≤ 0.005, **** *p* ≤ 0.001, ***** *p* ≤ 0.0005. All tests one-tailed. ^a^ *n* = 22; ^b^ *n* = 12.

## Data Availability

The datasets used and/or analyzed for the present paper can be made available upon completion of the larger project from which they were drawn or upon a reasonable request to the corresponding author.
